# Where the cerebral infarction meets child, be vigilant about patent foramen ovale: a case report

**DOI:** 10.3389/fneur.2024.1363867

**Published:** 2024-05-17

**Authors:** Zai-qiang Zhang, Jia-wang Ding

**Affiliations:** ^1^Department of Cardiology, The First College of Clinical Medical Sciences, China Three Gorges University, Yichang, Hubei, China; ^2^Institute of Cardiovascular Diseases, China Three Gorges University, Yichang, Hubei, China

**Keywords:** patent foramen ovale (PFO), cerebral infarction, paradoxical embolism, young child, case report

## Abstract

**Background:**

While cerebral infarction in children is rare, its prognosis is poor, and this condition can seriously burden society and families. A correlation between patent foramen ovale (PFO) and ischemic stroke has not been found in pediatric patients.

**Case presentation:**

We report a 7-year-old boy who suffered from multiple cerebral infarctions. Subsequently, the patient was diagnosed with an abnormal shunt of PFO. He underwent PFO closure and was followed up for 1 year. The patient did not experience any further cerebral infarction.

**Conclusions:**

With this case report, we want to illustrate that although the incidence rate of ischemic cerebral infarction in adolescents is very low, we should not neglect the role of PFO. Therefore, after exclusion other causes of cerebral infarction, PFO should be considered in adolescent and adult stroke patients with adult closure criteria in the same way.

## Background

Cerebral infarction occurs very rarely in adolescents, but the prognosis is usually poor. A patent foramen ovale (PFO) is a highly prevalent finding in cryptogenic ischemic stroke, particularly in adults, and the association between PFO and ischemic stroke has been documented in a large number of observational studies. However, this correlation has not been confirmed in pediatric patients. With the development of ultrasound technology, an increasing number of cases have been found to be related to paradoxical embolization through PFO and embolie strokes. Here, we report a young child with a cerebral infarction who presented with paradoxical embolism through PFO.

## Case presentation

**Figure 1:**
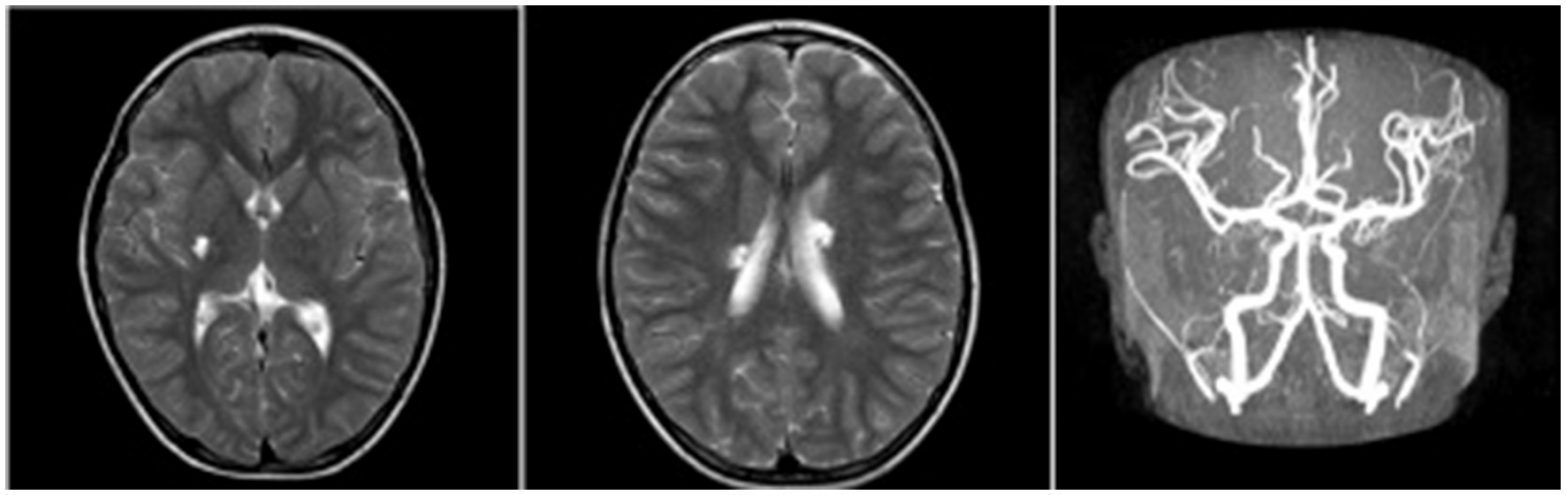
Craniocerebral MRI revealed multiple patchy abnormal signals in the bilateral paraventricular basal ganglia area and the right half oval center, and cerebral MRA showed no abnormalities.

**Figure 2:**
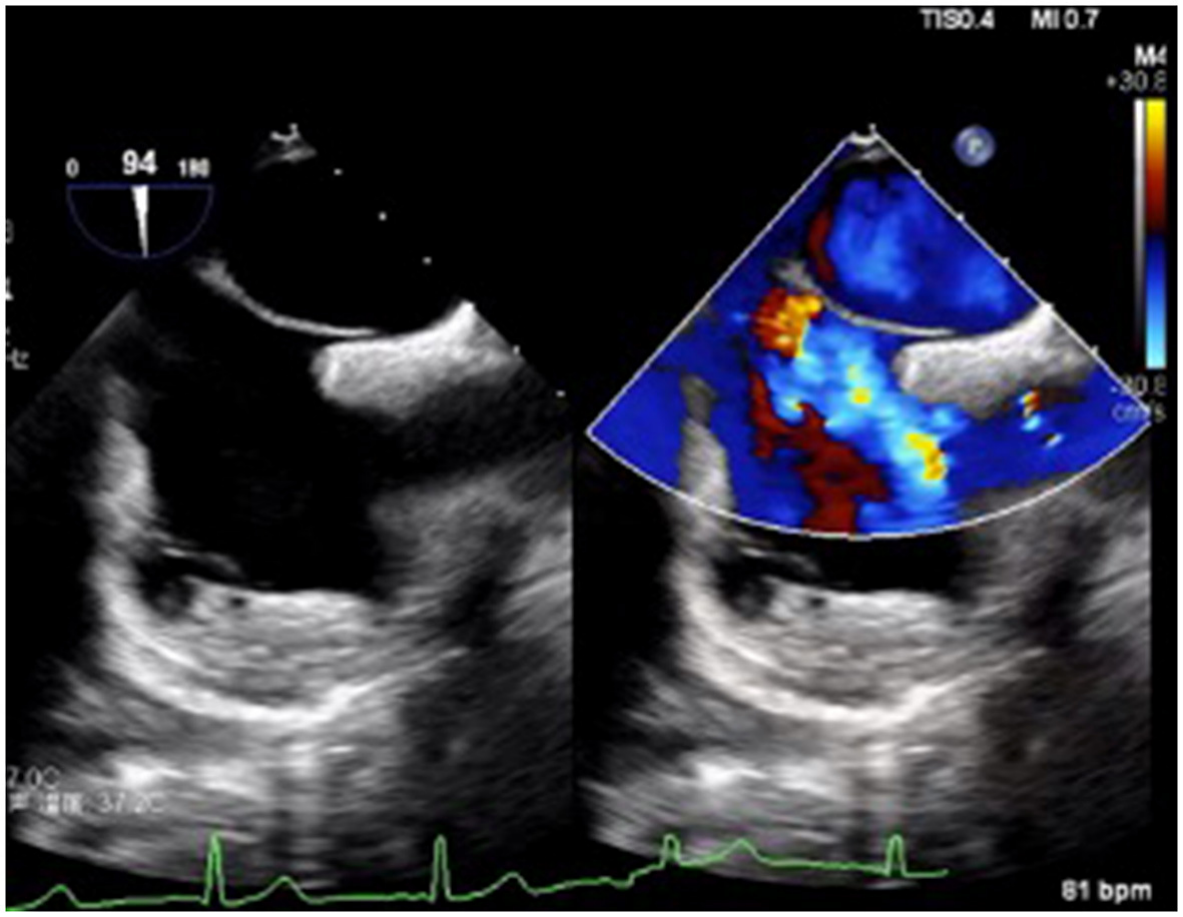
TEE revealed a foramen ovale of the atrial septum, with a width of ~2.2 mm and a length of ~15 mm.

**Figure 3:**
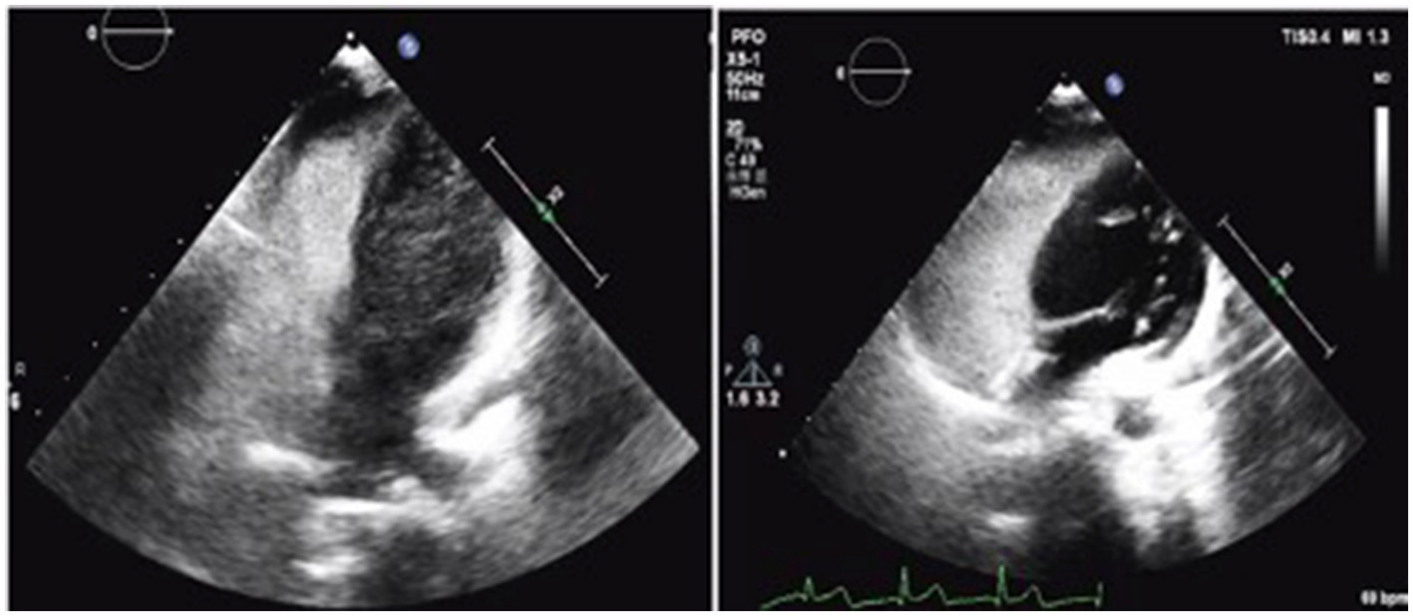
**(Left)** Before PFO closure, right ventricular contrast-enhanced ultrasound indicated positive results. **(Right)** After 6 months of PFO closure, the right ventricular contrast-enhanced ultrasound was negative, and the TCD foaming test was negative.

**Figure 4:**
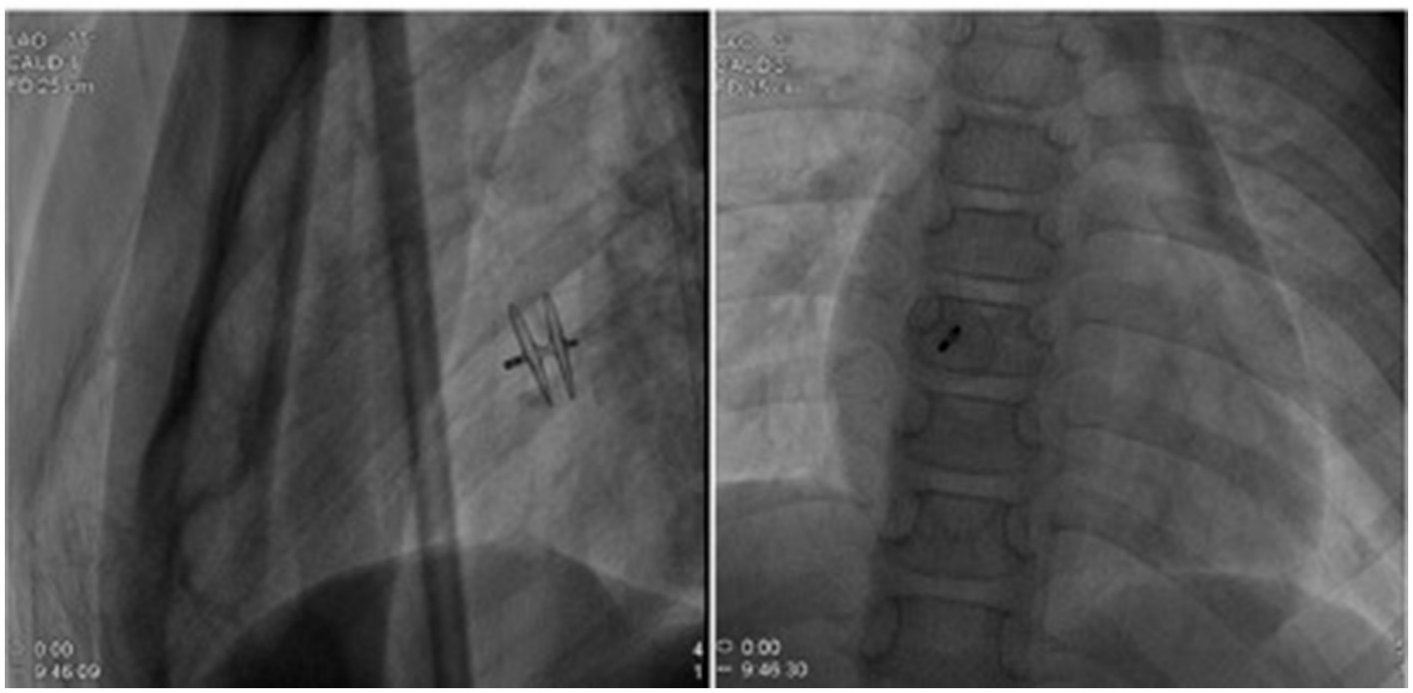
This procedure was performed with the implantation of an 18 mm Amplatzer PFO device.

A 7-year-old boy was referred to our center due to headache and left limb weakness. Craniocerebral magnetic resonance imaging revealed multiple patchy abnormal signals in the bilateral paraventricular basal ganglia area and the right half oval center. Softening lesions were considered, and no notable abnormalities were observed in the cerebral blood vessels (Figure 1). Transthoracic echocardiography indicated a right to left shunt signal with a width of ~2.5 mm in the middle of the atrial septum. Transesophageal echocardiography revealed a fissure-like echo at the foramen ovale of the atrial septum, with a width of ~2.2 mm and a length of ~15 mm (Figure 2). Right ventricular contrast-enhanced ultrasound indicates that a large amount of microbubble echoes were observed in the second cardiac cycle of the left heart in a resting state. During the Valsalva maneuver, a large amount of microbubble echoes were observed in the first cardiac cycle of the left heart (Figure 3, Left). The transcranial doppler (TCD) foaming experiment indicated that there was no microbubble echo in the resting state, and a large number of microbubble echoes were observed within 10 s during the Valsalva maneuver. Bilateral lower limb venous ultrasound showed no obvious thrombosis or plaque formation. The laboratory examination (included D-dimer) and electrocardiogram (ECG) showed no abnormalities. Inquire about the patient's medical history. The patient has no family history of coagulation disorders. A detailed medical history inquiry revealed that at the age of 2 years, the child experienced headaches, unclear language, and left limb weakness. Emergency cranial magnetic resonance imaging showed patchy and slightly longer T1 and T2 signal shadows in the bilateral basal ganglia area, with a larger range on the right side and a maximum cross-sectional size of approximately 1.5 × 1.2 cm. Bilateral abnormal signals in the basal ganglia area were observed, indicating demyelination changes. The relationship between recurrent cerebral infarction and abnormal shunting caused by PFO is considered in patients. The PFO closure decision was adopted after a detailed discussion. This procedure was performed under local anesthesia with the implantation of an 18 mm Amplatzer PFO device (Figure 4). The patient was discharged the next day. Antiplatelet therapy was administered 6 months after PFO closure. Follow-up after 1 year did not reveal any further symptoms of headache or limb weakness. After PFO closure, no new infarct was found on cranial MRI. The right ventricular contrast-enhanced ultrasound was negative, and the TCD foaming test was negative (Figure 3, Right).

## Discussion and conclusions

The foramen ovale is a separation between the septum primum and septum secundum at the anterior–superior portion of the septum, which is present in the fetus and enables the venous blood to bypass the non-functioning fetal lung and go directly to the left side of the heart. After birth, this communication closes in the majority of people but remains patent in 25–30% (1). The PFO, while frequently clinically silent and only open when the pressure increases on the right cardiac cavities, such as during a Valsalva maneuver, can exert pathological effects by allowing thrombi to transit from the venous to the arterial circulation (paradoxical embolization) (2).

Among adolescent patients, the incidence rate of arterial ischemic stroke (AIS) is estimated to be 0.6 per 100,000, indicating an important cause of morbidity and mortality among adolescents, and the risk of recurrence is also high (3). In these patients, the cause of cardiac embolism was discovered in 15%, with 50% suffering from PFO (3). In adults, the correlation between cryptogenic stroke and PFO has been validated. Multiple studies have shown that PFO closure can significantly reduce the risk of cryptogenic AIS recurrence compared to medical antithrombotic therapy (4–6). However, the pathogenic role of PFO in childhood remains controversial. The pediatric guidelines only suggest that cryptogenic stroke may benefit from PFO closure.

Our case shows multiple cryptogenic strokes in a 7-year-old boy, possibly due to an abnormal shunt of PFO. The etiology of stroke in children is complex, and the common diseases of ischemic stroke in children include congenital heart malformations, sickle cell disease, infections, and collagen tissue abnormalities (7). However, nearly half of these cases are found in previously healthy children, known as cryptogenic stroke. In adults, the mechanisms of PFO causing ischemic stroke mainly include paradoxical embolism, *in situ* thrombosis, and arrhythmias. Paradoxical embolism is the most common mechanism, and embolism from the venous system or right atrium can directly access the arterial system through PFO, leading to ischemic stroke (8). *In situ* thrombosis refers to the formation of thrombi in the tunnel of PFO, and some studies suggest that PFO patients in a hypercoagulable state have a higher risk of stroke (9). There are also some reports of thrombi attached to the atrial septum and foramen ovale (10). Previous studies have found that PFO can alter left atrial electrical activity and easily lead to the occurrence of some atrial arrhythmias (11). In addition, Hanley et al. observed that some high-risk foramen ovale are associated with the occurrence of atrial fibrillation (AF) (12). There is currently insufficient clinical evidence to demonstrate the correlation between PFO and stroke in pediatric stroke patients compared to adult stroke patients. However, there are still some small studies indicating that cryptogenic stroke may benefit from PFO closure (13).

To summarize, with this case report, we want to illustrate that PFO meeting the adult criteria for closure should be considered the same way in adolescents as in adult stroke patients after careful evaluation to rule out other causes of stroke (including hypercoagulable state, other sources of cardiac embolism, and intracranial and extracranial arterial lesions including dissection). There was no significant difference in safety and effectiveness of this treatment.

## Data availability statement

The raw data supporting the conclusions of this article will be made available by the authors, without undue reservation.

## Ethics statement

The studies involving humans were approved by Ethics Committee of Yichang Central People's Hospital. The studies were conducted in accordance with the local legislation and institutional requirements. Written informed consent for participation in this study was provided by the participants' legal guardians/next of kin. Written informed consent was obtained from the individual(s), and minor(s)' legal guardian/next of kin, for the publication of any potentially identifiable images or data included in this article.

## Author contributions

Z-qZ: Writing – review & editing, Writing – original draft. J-wD: Writing – review & editing.
